# A 3 month nutrition and exercise program improved hallux strength among senior daycare center users in Korea: a cluster randomized controlled trial

**DOI:** 10.3389/fpubh.2024.1364908

**Published:** 2024-07-19

**Authors:** Jiwon Sim, Jongguk Lim, Hayoung Lee, Sohyun Park, Dongsoo Shin

**Affiliations:** ^1^Department of Food Science and Nutrition, Hallym University, Chuncheon, Republic of Korea; ^2^The Korean Institute of Nutrition, Hallym University, Chuncheon, Republic of Korea; ^3^Department of General Physical Education, Ilsong Liberal Arts School, Hallym University, Chuncheon, Republic of Korea; ^4^School of Nursing, Hallym University, Chuncheon, Republic of Korea; ^5^Areumdeurinamu Children Hospital, Sejong, Republic of Korea

**Keywords:** senior daycare center, intervention study, dietary supplements, exercise, aged, hallux

## Abstract

**Introduction:**

With a growing aging population, the focus on the health and well-being of older adults, especially in preventing falls, becomes crucial. This 3 month study, initiated in July 2022, aimed to assess the impact of a nutrition and exercise program in senior daycare centers in Chuncheon, South Korea.

**Methods:**

A 3 month study, beginning in July 2022, included 204 older adults from 10 senior daycare centers in Chuncheon, South Korea. Randomly assigned to intervention or control groups, the intervention involved nutrition, daily toe exercises, or both. Control centers received interventions post-measurements. Pre- and post-intervention analyses used paired t-tests and multiple linear regression, assessing metrics like toe grip strength for significance. While 204 were initially enrolled, the analysis included 151 participants due to dropouts.

**Results:**

Participants, with a mean age of 83.3 years (43.1% aged ≥ 85 years), exhibited mild to moderate cognitive impairment and multiple chronic illnesses. Health data indicated that 37.3% were obese, and the average BMI was 24.0 kg/m^2^. Both the intervention and control groups showed significant improvements in toe grip strength post-intervention. Specifically, the exercise-only and combined exercise-nutrition groups demonstrated significant differences in hallux strength compared to the control group after adjusting for age and gender.

**Conclusion:**

The study showed that a basic nutrition and exercise program increased toe strength in older adults with chronic diseases, including mild cognitive impairments. This intervention holds potential to prevent muscle strength decline and reduce fall risks in older individuals. As the first of its kind in Korean senior daycare centers, it emphasizes the need for future research and standardized programs for senior daycare users.

## 1 Introduction

Many countries are experiencing rapid aging because of increased life expectancy and a declining birthrate (1). In South Korea (hereafter, Korea), the transition to an aging society began in 2000, when the population aged ≥ 65 reached 7%. This was followed by a rapid transition to a society in which 14% of the population were older adults in just 18 years (2). Projections indicate that Korea will become a super-aged society by 2024, in which approximately 20% of the population will be people aged ≥ 65 years (3). This aging rate is the fastest among Organization for Economic Cooperation and Development countries, underscoring the seriousness of Korea’s aging problem (4).

Older adult health has become a critical area of focus as the proportion of the population that is aging continues to increase. The decline in the health of older adults not only significantly reduces their quality of life but also affects the national economy caused by increased medical expenses. Health insurance statistics show that in 2021, medical expenses for older people reached 41.3829 trillion won, a jump of approximately 1.5 times compared with that in 2017, accounting for approximately 43% of total medical expenses (3). As people age, their metabolism and physical function decline, leading to various health problems, of which sarcopenia is a common one. Sarcopenia, characterized by a significant decrease in skeletal muscle mass and function in older people, has been reported to lead to adverse outcomes such as falls, reduced mobility, fractures, exacerbation of chronic diseases, and increased mortality, and as aging progresses, it has emerged as a significant global health issue (5–8). The reduction in muscle mass due to aging in older adults leads to a decrease in toe muscle strength and function (9, 10). According to the previous research analyzing toe muscle strength in participants of different ages, it was observed that toe strength significantly decreases with increasing age (11, 12). Toe muscles influence walking speed, maximum walking distance, and the movements required for walking, as well as contribute to maintaining balance (13–15). Furthermore, the muscles in the feet play a significant role in the movement and balance function of the legs, and toe strength serves as a useful indicator of the risk of falls, reflecting lower limb muscle strength (16–19). This is essential for maintaining stable standing and walking (20, 21). Therefore, the decline in toe function and strength due to muscle loss is highly associated with the risk of falls (18, 22, 23). Accordingly, previous research on fall prevention in older adults has emphasized the importance of preventing muscle loss and enhancing muscle strength (24, 25). Furthermore, a previous study comparing toe strength and the occurrence of falls in older and middle-aged individuals confirmed a higher incidence of falls in the group with lower toe strength (26–28). Additionally, declining toe muscle strength can lead to toe deformities and impairments (29, 30), which are quite common among older adults (31, 32). Toe deformities result in walking impairments and an increased risk of falls (22, 33–35). In a study investigating the relationship between foot problems and balance and walking function in older adults aged between 75 and 93 years, it was found that individuals with foot issues exhibited significantly lower balance and walking function (34). The decline in toe muscle strength and function due to aging has a negative impact on the health and safety of older adults. Therefore, research on strength and toe muscle strength enhancement is needed to prevent falls and promote health in the older adults.

Numerous studies emphasize that regular physical exercise provides various health benefits during the aging process. First, appropriate physical exercise enhances cognitive functions in older adults, improving memory, thinking speed, and reasoning abilities, thereby contributing to an overall better quality of life (36). Additionally, exercise helps prevent sarcopenia by maintaining or increasing muscle mass and strength, which prevents the decline in physical function in the older adults (37). Furthermore, it improves balance and mobility, reducing the risk of falls, and maintains cardiovascular health, thereby lowering the risks of heart disease, hypertension, and stroke (38). From a mental health perspective, exercise alleviates symptoms of depression and anxiety, promoting emotional well-being. These benefits highlight the critical role that regular exercise plays in enhancing the health, safety, and overall well-being of older adults. Nutrition also plays a crucial role in improving the health of older adults. Protein intake is essential for the prevention and treatment of sarcopenia. Adequate protein consumption helps maintain or increase muscle mass, improves muscle strength, and supports overall physical function (39). Older adults tend to have increased protein requirements due to decreased metabolism and absorption efficiency. A protein-rich diet can reduce muscle loss, enhance physical function, and improve overall health status (40). Therefore, proper nutrition, especially adequate protein intake, is vital for maintaining and improving the health of older adults.

A senior daycare center provides services to older individuals who require daily care. Older adults who can maintain a certain level of functioning can use these services during the day and remain in their homes and communities. Therefore, daycare centers are important in the community as places where “aging in place (AIP)” can be realized. AIP refers to older people spending their later years in the same place where they have always lived and where they have always performed their daily living activities. This not only improves the quality of life for the individual older person but it is also being presented as a positive alternative from a national policy perspective (41). However, most of these facilities lack nutrition and exercise professionals, resulting in inadequate nutrition and exercise programs (42). In addition, older adults who use daycare centers often suffer from chronic conditions such as dementia, which increases their risk for falls and potential injuries (43).

Therefore, proactive policies and research targeting these groups at high risk for falls are needed in the context of senior daycare centers. However, significantly no study has examined fall prevention and promotion of the overall health of seniors attending these daycare centers. Thus, this study aimed to evaluate the effectiveness of a health-promotion program that included a balanced diet and toe strength exercise to improve the toe grip strength. The novelty of this study lies in its focus on a comprehensive health promotion program specifically tailored for older adults in senior daycare centers, incorporating both nutritional and physical exercise components. This approach addresses the dual aspects of nutritional balance and muscle strengthening, with a particular focus on toe strength, especially hallux strength, which is crucial for maintaining balance and preventing falls. By evaluating the combined impact of diet and toe strength exercise, this study offers a unique perspective on fall prevention and overall health improvement in a vulnerable population that has been largely overlooked in previous research.

This study demonstrates the potential to improve the quality of life for older adults. It offers effective health promotion strategies that can be practically applied in senior daycare centers, making it highly relevant for healthcare providers, policymakers, and caregivers. Furthermore, the study contributes to public health goals by reducing healthcare costs associated with chronic diseases and fall-related injuries among the older adults. By proving the effectiveness of these interventions, the research highlights the necessity for policies and practices aimed at enhancing the health of the aging population. Additionally, this study provides a comprehensive approach that can be adapted and implemented across various care settings.

## 2 Materials and methods

### 2.1 Study design, recruitment, and randomization

A cluster randomized controlled trial was conducted in senior daycare centers in Chuncheon, Korea. An initial outreach to 30 centers was made using the contact information provided by the City of Chuncheon. Among these, 13 centers expressing interest were involved in a random selection process conducted where researchers and key personnel from the centers were present. Subsequently, three centers were excluded from the intervention, owing to limited resources and sample size exceeding the planned limit.

The recruitment size was determined using G-power 3.1.9.2 (Dusseldorf University, Germany). Considering a significance level of 0.05, effect size of 0.5, power of 80%, each group required a minimum of 34 participants. To account for a potential dropout rate of 30%, 174 participants were targeted for recruitment. Since participants were recruited in groups from participating centers, a total of 204 participants participated in the pre-intervention assessment.

The 10 centers were assigned into four intervention arms: nutrition group, which received protein-enriched supplemental beverages and nutrition education for both staff and senior users; exercise group, engaging in guided daily toe exercises; nutrition + exercise group, participating in both nutrition and exercise protocols; and control group, which received delayed intervention components after the post-intervention measurements.

Data collection began in June 2022, followed by the development of specific intervention protocols appropriate for the participating centers and participants. The intervention program was implemented in 3 months beginning in July 2022, followed by post intervention assessments.

The specific research methods employed are outlined below. This study was approved by the Institutional Review Board of Hallym University (HIRB-2022-021-2-RR).

### 2.2 Participants

In total, 630 older people who frequented the 10 participating centers were invited to assess their eligibility for the study. The selection criteria for participation were delineated to ensure relevance to the study’s objectives and were as follows: willingness to participate in the study, age ≥ 65 years, capability to communicate effectively and sufficient physical function to engage in the program, and attendance at the center for a minimum of 3 days per week. Out of the initial cohort of 630 invited individuals, 420 (66.7%) did not meet the inclusion criteria, and an additional 6 (1.0%) declined to participate. Consequently, 204 individuals (32.4%) were deemed eligible and participated in the pre-intervention survey.

**Figure 1:**
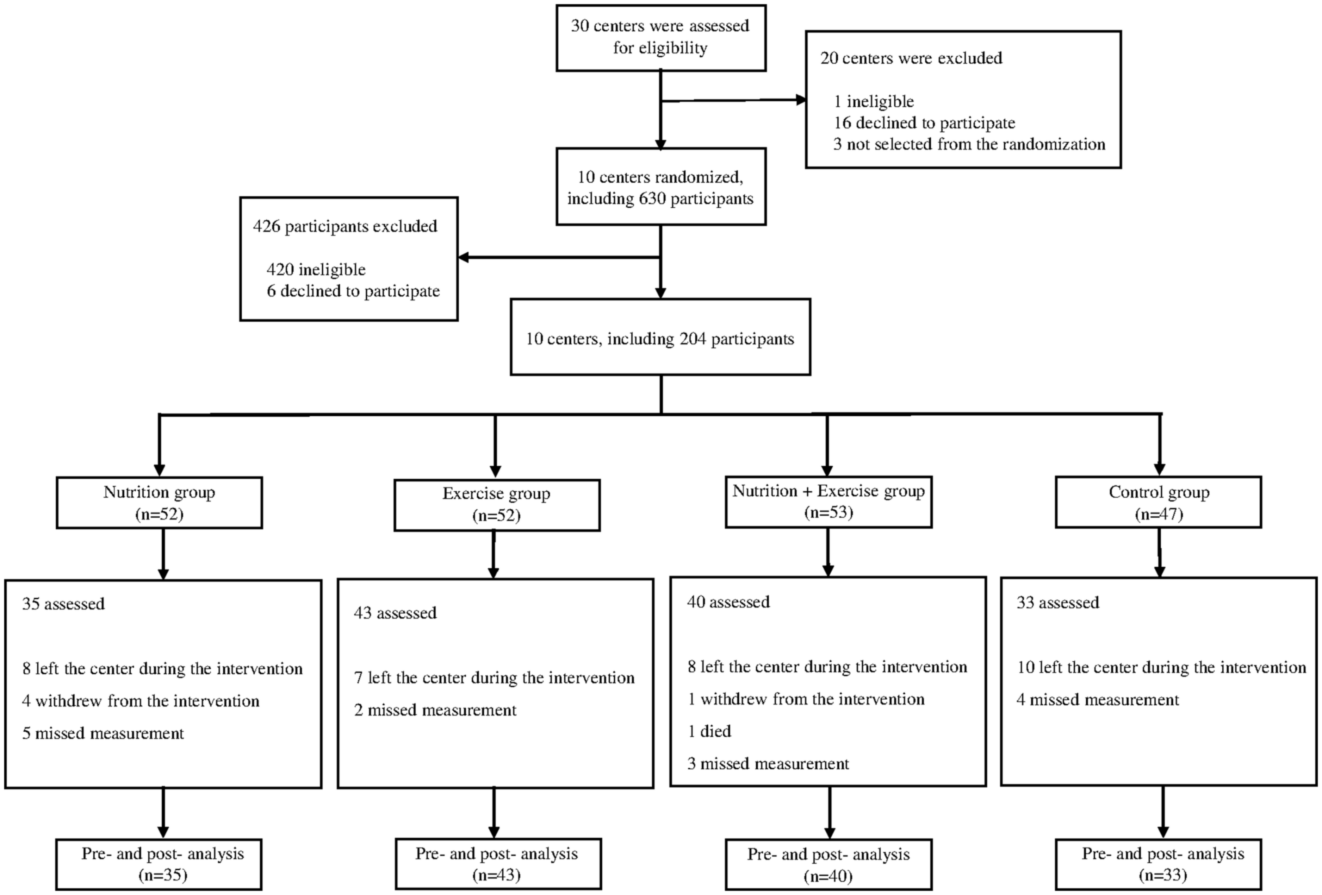
Flow chart of study participations and intervention assignment.

During the 3-month intervention period, participant dropout was noted across the groups, with 17 (32.7%), 9 (17.3%), 13 (24.5%), and 14 (29.8%) participants from the nutrition, exercise, nutrition + exercise, and control groups, respectively. The primary reason for this dropout was hospitalization or admission to a nursing home, accounting for 33 (62.3%) participants. Subsequent reasons for dropout included missing measurements for 14 (26.4%) participants, discontinuation of the intervention for 5 (9.4%), and death of 1 (1.9%). The major causes for the missing measurements were absence, health-related inability to participate in the measurement, and refusal to the measurement. The discontinuation of the intervention was specifically related to the provision of nutritional supplements, with reasons including side effects such as abdominal pain and diarrhea and intake refusal (Figure 1).

### 2.3 Pre-post measurements

The pre-intervention measurements were conducted in June 2022 for over a month. Researchers assessed total toe strength, hallux strength, sociodemographic characteristics, and health indicators in the participating daycare centers. The total toe strength was measured using a T.K.K.3362 toe grip dynamometer (Takei Scientific Instruments, Niigata, Japan), a validated tool in previous studies that established the correlation between toe strength and walking ability (44–47). Hallux strength, which determines the overall toe strength level (45, 48), was gauged using a newly developed portable device, which was designed in collaboration with the university’s makerspace. The device’s error rate is within 0.05%, which meets international standards (49). Following proper posture guidelines, measurements were taken with participants seated and socks removed. Height was obtained from care center records, and weight was measured during the assessment. The body mass index (BMI) was calculated from pre-intervention height and weight measurements (as kg/m^2^). BMI values were categorized as underweight (BMI < 18.5), normal weight (18.5–22.9), overweight (23.0–24.9), and obese (BMI ≥ 25.0) (50). A survey questionnaire was administered by trained data collectors. The post-intervention measurements were conducted similarly, beginning in October, and lasted 1 month after the 3 month intervention period.

### 2.4 Intervention components

The interventions were divided into nutrition and exercise programs. These programs were designed according to the operational characteristics and usage patterns of the senior daycare center, as identified through the preliminary interviews with key stakeholders and senior service users (results not presented here). The main components of the interventions are presented in Table 1.

**Table 1:** Summary and implementation timeline of the intervention components for each group.^*^

	Nutrition group	Exercise group	Nutrition + exercise group	Control group
June	Pre-intervention survey and measurement			
July	Nutrition supplementation Nutrition education (3 sessions)	Provision of exercise equipment and demonstration sessions	Nutrition supplementation Nutrition education (3 sessions) Provision of exercise equipment and demonstration sessions	
August	Nutrition supplementation Nutrition education (2 sessions) Provision of nutrition information (posters)	Exercise demonstration sessions Provision of posters with exercise photos	Nutrition supplementation Nutrition education (2 sessions) Provision of nutrition information (posters) Exercise demonstration Provision of posters with exercise photos	
September	Nutrition supplementation Provision of nutrition information (via mobile phones)	Exercise demonstration sessions	Nutrition supplementation Provision of nutrition information (via mobile phones) Exercise demonstration sessions	
October	Nutrition supplementation Provision of nutrition information (via mobile phones)	Exercise demonstration sessions	Nutrition supplementation Provision of nutrition information (via mobile phones) Exercise demonstration sessions	
November	Post-intervention survey and measurement			
December				Nutrition supplementation Provision of exercise equipment and demonstration sessions

^*^The intervention was conducted for a duration of 3 months, from 11th July to 11th October.

#### 2.4.1 Nutrition

The nutrition groups were provided with supplemental drinks containing 20 g of protein per 200 mL serving, 5 days a week for 3 months to address challenges in improving meal quality due to professional and budget constraints. Consumption records were maintained by center staff, and participants with adverse symptoms or refusal were excluded. A targeted nutrition education program for center employees was developed, consisting of weekly 2-h sessions at the university over 4 weeks. The education program included lectures and cooking practice sessions conducted by professionals, covering topics such as “nutritional characteristics of older adults,” “dysphagia,” “dietary guidelines for the older adults,” and “cooking practice for the older adults.” Additionally, visually tailored dietary guideline posters were provided. These materials were developed under expert guidance and aligned with relevant government guidelines. Posters were created separately for staff and older adults participants; staff posters included detailed information on recommended nutrient intake and dietary guidelines for the older adults, while posters for the older adults featured simple, image-based dietary and hydration guidelines. It was recommended that these posters be placed in highly visible locations within the center. Lastly, informative emails containing text and images on topics such as nutritional guidelines and general food safety tips were sent to the center staff over 4 weeks. It was recommended that these emails be utilized for staff training, education for the older adults, and newsletters to families.

#### 2.4.2 Exercise

The exercise program was designed for seated participants, incorporating simple movements with the body or tools such as soft exercise balls and towels, tailored to their low cognitive function and physical strength. A minimum of 30 min per session, three times weekly, was recommended. The routine began with breathing and stretching, followed by activities such as writing names with toes, rolling balls with toes, and lifting towels with toes, and ended with toe stretching. Experts initially demonstrated the program and then trained the care center’s employees, who conducted the exercises and documented participation. Guidelines with photos and explanations were provided, and personal visits were made to centers with low participation rates to ensure understanding and motivation.

### 2.5 Statistical analysis

All participants who were randomized were included in the analyses except for dropouts, irrespective of their compliance level, as long as pre- and post-measurement outcome data were available (51). The data from 151 participants were used in the final analyses for this manuscript. To monitor compliance, participants were required to maintain a daily log confirming their adherence to the program activities. All the participants in the intervention groups that are included in the final analyses reached more than 80% of compliance level of their assigned intervention components. Descriptive statistics such as means and standard deviations of the variables were calculated, and chi-square tests were performed. The paired *t*-test was conducted when complete pre- and post-intervention measurements were available. Further, a multiple regression analysis was conducted to compare toe strength changes between groups before and after the program, adjusting for age and sex. A significance level of 0.05 was set for all analyses. Data were analyzed using Stata 17.0.

## 3 Results

The general characteristics of the study participants are presented in Table 2. The mean age of the participants was 83.3 years, and 88 (43.1%) participants were classified as late older people, i.e., aged ≥ 85 years. In the comparison of age groups, the nutrition group had a significantly higher average age (85.3 years) than the other groups. Regarding educational attainment, 69 (40.8%) participants had no formal education, 63 (37.3%) had completed either elementary or middle school, and 37 (21.9%) had completed high school or higher education. As for alcohol consumption, 17 (10.1%) participants reported drinking regularly. Moreover, 145 (86.8%) participants reported never having smoked, 15 (9.0%) reported past smoking, and 7 (4.2%) were identified as current smokers. Out of all the participants, 75 (37.3%) were classified as obese, 71 (35.3%) as normal weight, 39 (19.4%) as overweight, and 16 (8.0%) as underweight. The average BMI of all participants was 24.0 kg/m^2^. On average, the participants had two chronic diseases and took 4.6 medications per day. Regarding dental health, 79 (47%) participants used dentures, and 46 (27.4%) reported experiencing pain when chewing. Smoking status, alcohol intake, and dental health were included as these factors can affect overall health. Smoking and alcohol consumption are risk factors for chronic diseases, and poor dental health can impact nutrition. These variables help provide a comprehensive understanding of participants’ health and account for potential confounding factors in the analysis.

**Table 2:** General characteristics of the study participants by intervention groups.^*^

	Control	Nutrition	Exercise	Nutrition + exercise	Total	*p*-value
**Sex, *n* (%)**						
Men	15 (31.9)	15 (28.9)	15 (28.9)	15 (28.3)	60 (29.4)	0.979
Women	32 (68.1)	37 (71.2)	37 (71.2)	38 (71.7)	144 (70.6)	
**Age**						
Mean ± SD	82.2 ± 6.4	85.3 ± 5.7	83.4 ± 5.3	82.3 ± 6.1	83.3 ± 6.0	0.030
65–84 years, *n*(%)	32 (68.1)	23 (44.2)	31 (59.6)	30 (56.6)	116 (56.9)	0.114
≥85 years	15 (31.9)	29 (55.8)	21 (40.4)	23 (43.4)	88 (43.1)	
**Education level, *n* (%)**						
No formal schooling	18 (41.9)	13 (41.9)	22 (44.0)	16 (35.6)	69 (40.8)	0.673
Elementary/middle school	15 (34.9)	13 (41.9)	20 (40.0)	15 (33.3)	63 (37.3)	
High school or higher	10 (23.3)	5 (16.1)	8 (16.0)	14 (31.1)	37 (21.9)	
**BMI, *n* (%)** ^**+**^						
Mean ± SD	23.6 ± 3.7	24.2 ± 4.2	24.6 ± 4.8	23.7 ± 3.4	24.0 ± 4.1	0.537
Underweight	3 (6.5)	5 (10.0)	5 (9.6)	3 (5.7)	16 (8.0)	0.097
Normal	16 (34.8)	12 (24.0)	19 (36.5)	24 (45.3)	71 (35.3)	
Overweight	14 (30.4)	13 (26.0)	4 (7.7)	8 (15.1)	39 (19.4)	
Obesity	13 (28.3)	20 (40.0)	24 (46.2)	18 (34.0)	75 (37.3)	
**Care level, *n* (%)** ^**‡**^						
Levels 1–2	3 (6.4)	3 (5.8)	1 (1.9)	1 (1.9)	8 (3.9)	0.412
Levels 3–4	24 (51.1)	36 (69.2)	34 (65.4)	35 (66.0)	129 (63.2)	
Levels 5–6	20 (42.6)	13 (25.0)	17 (32.7)	17 (32.1)	67 (32.8)	
**Drinking status, *n* (%)**						
No	39 (88.6)	29 (93.6)	45 (90.0)	38 (88.4)	151 (89.9)	0.887
Yes	5 (11.4)	2 (6.5)	5 (10.0)	5 (11.6)	17 (10.1)	
**Smoking status, *n* (%)**						
Never smoker	32 (72.7)	29 (96.7)	43 (86.0)	41 (95.4)	145 (86.8)	0.006
Ex-smoker	6 (13.6)	1 (3.3)	6 (12.0)	2 (4.7)	15 (9.0)	
Current smoker	6 (13.6)	0 (0.0)	1 (2.0)	0 (0.0)	7 (4.2)	
**Denture status, *n* (%)**						
No	20 (50.0)	19 (50.0)	27 (60.0)	23 (51.1)	89 (53.0)	0.745
Yes	20 (50.0)	19 (50.0)	18 (40.0)	22 (48.9)	79 (47.0)	
**Having difficulty in chewing, *n* (%)**						
No	35 (79.6)	18 (62.1)	38 (76.0)	31 (68.9)	122 (72.6)	0.349
Yes	9 (20.5)	11 (37.9)	12 (24.0)	14 (31.1)	46 (27.4)	
**Number of chronic diseases** ^**¶**^						
Mean ± SD	2.2 ± 1.0	2.0 ± 1.0	1.8 ± 0.8	2.0 ± 1.1	2.0 ± 1.0	0.133
**Number of medications** ^**¶**^						
Mean ± SD	5.4 ± 4.1	3.9 ± 4.1	5.1 ± 4.0	4.1 ± 4.3	4.6 ± 4.2	0.316

^*^Because of missing values, the sample size is not the same for all variables. ^+^BMI = Body mass index; weight(kg)/height(m)^2^, Underweight, < 18.5; normal, 18.5≤~<22.9; overweight, 23.0≤~<24.9; obesity, ≥25.0. ^‡^Care level is a grading system for older adults, ranging from 1 to 6, which assesses their ability to perform daily activities independently. Lower grades indicate higher dependency and the need for more comprehensive care due to age-related conditions. ^¶^Number of chronic diseases and medications were counted based on the medical records and prescription lists provided by the centers and participants.

Table 3 presents a comparison of BMI, total toe grip strength, and hallux strength values before and after the intervention within each intervention arm. Their BMI did not change after the intervention. The total toe grip strength significantly increased in the nutrition (1.0 kg, *p* = 0.024), exercise (2.0 kg, *p* < 0.001), and control (1.2 kg, *p* = 0.003) groups. Hallux strength significantly rose across all groups, including the nutrition (1.9 kg, *p* < 0.001), exercise (2.3 kg, *p* < 0.001), nutrition + exercise (2.2 kg, *p* < 0.001), and control (1.0 kg, *p* = 0.029) groups.

**Table 3:** Comparing pre- and post-intervention measurements using the paired t-test.^*^

		Mean(SD)		
		Pre^+^	Post^‡^	*P*-value^§^
BMI^¶^ (kg/m^2^) (*n* = 151)	Control (*n* = 33)	23.2 (3.7)	23.6 (3.9)	0.153
	Nutrition (*n* = 35)	24.6 (4.2)	24.7 (4.1)	0.439
	Exercise (*n* = 43)	24.6 (4.6)	24.9 (4.6)	0.349
	Nutrition + Exercise (*n* = 40)	23.8 (3.4)	24.3 (3.9)	0.124
Total toes grip strength (kg) (*n* = 144)	Control (*n* = 31)	2.6 (2.0)	3.8 (2.4)	0.003
	Nutrition (*n* = 34)	2.8 (2.3)	3.8 (2.7)	0.024
	Exercise (*n* = 40)	3.2 (2.8)	5.2 (3.4)	<0.001
	Nutrition + Exercise (*n* = 39)	2.6 (1.4)	3.2 (2.2)	0.080
Hallux strength (kg) (*n* = 145)	Control (*n* = 32)	3.2 (1.8)	4.2 (2.8)	0.029
	Nutrition (*n* = 33)	3.0 (1.7)	4.9 (2.5)	<0.001
	Exercise (*n* = 40)	3.4 (2.2)	5.7 (3.6)	<0.001
	Nutrition + Exercise (*n* = 40)	2.6 (1.4)	4.8 (2.6)	<0.001

^*^Because of missing values, the sample size is not the same for all variables. ^+^Pre-intervention measurement. ^‡^Post-intervention measurement. ^§^The *P*-value indicates the level of statistical significance for the differences in pre- and post-intervention mean values. ^¶^BMI, Body mass index.

Table 4 summarizes the changes in BMI, total toe grip strength, and hallux strength before and after the intervention compared with the control group, with multiple regression models adjusted for age and sex. The outcome variable in the regression analysis was the difference between post- and pre-intervention measurements. Across all intervention arms, no significant differences were noted in BMI and total toe grip strength when compared with the control group. However, regarding hallux strength, statistically significant differences were observed in the exercise group, with a difference of 2.3 kg (*p* = 0.034), and the nutritional + exercise group, with a difference of 2.2 kg (*p* = 0.033), compared with the control group.

**Table 4:** Regression analysis of pre- and post-intervention differences compared with the control group.^*^

		Mean(SD)			
		Pre^+^	Post^‡^	Difference^§^	*P*-value^¶^
BMI^**^ (kg/m^2^) (*n* = 151)	Control (*n* = 33)	23.2 (3.7)	23.6 (3.9)	0.4 (1.6)	-
	Nutrition (*n* = 35)	24.6 (4.2)	24.7 (4.1)	0.1 (1.0)	0.339
	Exercise (*n* = 43)	24.6 (4.6)	24.9 (4.6)	0.2 (1.7)	0.597
	Nutrition + Exercise (*n* = 40)	23.8 (3.4)	24.3 (3.9)	0.5 (1.9)	0.869
Total toes grip strength (kg) (*n* = 144)	Control (*n* = 31)	2.6 (2.0)	3.8 (2.4)	1.2 (2.0)	-
	Nutrition (*n* = 34)	2.8 (2.3)	3.8 (2.7)	1.0 (2.5)	0.531
	Exercise (*n* = 40)	3.2 (2.8)	5.2 (3.4)	2.0 (2.4)	0.100
	Nutrition + Exercise (*n* = 39)	2.6 (1.4)	3.2 (2.2)	0.6 (2.1)	0.373
Hallux strength (kg) (*n* = 145)	Control (*n* = 32)	3.2 (1.8)	4.2 (2.8)	1.0 (2.4)	-
	Nutrition (*n* = 33)	3.0 (1.7)	4.9 (2.5)	1.9 (2.1)	0.296
	Exercise (*n* = 40)	3.4 (2.2)	5.7 (3.6)	2.3 (3.5)	0.034
	Nutrition + Exercise (*n* = 40)	2.6 (1.4)	4.8 (2.6)	2.2 (2.6)	0.033

^*^Because of missing values, the sample size is not the same for all variables. ^+^Pre-intervention measurement. ^‡^Post-intervention measurement. ^§^Difference between pre- and post-measures in each group. ^¶^The *p*-values were obtained using regression analysis after controlling for age and sex. The outcome variable was the difference between post- and pre-intervention measurements, and the control group served as the reference group. ^**^BMI, Body mass index.

## 4 Discussion

To the best of authors’ knowledge, this study is the first to examine toe grip strength in older adults enrolled in Korean senior daycare centers. A 3 month program combining nutrition and exercise interventions was implemented for older adults in senior daycare centers. Results showed significant increases in both total toe grip strength and hallux strength across the nutrition, exercise, and control groups. In addition, significant differences in hallux strength were found between the exercise and nutrition + exercise groups compared with the control group, even after adjusting for age and sex. Toe grip strength, including hallux strength, could help older adults avoid falls (16–19), and older people in senior centers may benefit from nutrition and exercise programs.

The nutritional and exercise interventions implemented in this study were designed based on the characteristics of older adults individuals utilizing senior daycare centers. As part of the nutritional intervention, protein drinks containing a daily intake of 20 g of protein were provided. This amount satisfies approximately 40% of the recommended protein intake for the older adults, as proposed by the Korean Nutrition Society in 2015 (52). In addition, for older adults attending senior daycare centers, it was challenging to anticipate the effectiveness of nutritional education due to cognitive impairment. Therefore, nutritional education was conducted targeting senior daycare center staff. In a previous study that conducted nutritional education for staff in dementia wards, it was observed that the nutritional levels and dietary intake of older adults in the ward increased after the staff received nutritional education. This approach aligns with findings from previous research, demonstrating the indirect benefits of staff-directed nutritional interventions on the dietary habits of older adults (53). Furthermore, regarding the exercise intervention conducted in this study, prior research has established a connection between toe strength and falls. In a randomized controlled intervention study with individuals aged 60 and older, a 3 month toe exercise program resulted in a significant increase in toe muscle strength for the participating group compared to the control group. These findings are consistent with our results, which also indicate a significant improvement in toe muscle strength following the intervention (15).

In addition, studies that have focused on nutrition interventions, including the distribution of nutritional supplements, led to improvements in physical function (54) and frailty (55). Programs combining both nutrition and exercise have shown greater benefits (56, 57). Older participants who received exercise and nutrition interventions reported more positive effects on muscle mass than those who only received exercise intervention (58). Participants’ physical function improved after a 6-month program that included both nutrition and exercise interventions for older adults (59, 60). Furthermore, a study that assessed fall risk in older adults based on their muscle strength and physical function showed a decrease in the fall risk level following the intervention (61, 62). Thus, the nutrition and exercise program may reduce the risk of falls in older adults. Moreover, muscle strength improved among participants with mild cognitive impairment, which suggests early active intervention for older adults with declining cognitive function.

In this study, a greater difference in hallux strength than in total toe strength was observed between the groups. These results may be explained by the difficulties experienced in measuring total toe strength in an older population, particularly those with limited mobility and mild cognitive impairment. From our observations during data collection, many participants had more difficulty grasping five toes simultaneously than pushing one big toe. This may be due to cognitive impairments that hinder their ability to use their toes freely or the stiffness in their lesser toes resulting from a lack of appropriate stretching and movements. Thus, the use of the total toe grip strength among the older population poses a challenge (45, 46). We believe that this is one of the reasons for the insignificant increase in toe grip strength among nutrition + exercise group in post-intervention measurement. Some participants in this combined intervention group had a particularly hard time grasping five toes. Therefore, measuring the pressure of one big toe may be sufficient or even more feasible for measuring lower limb strength in very older populations with cognitive impairment. Furthermore, the hallux has demonstrated the strongest correlation with total toe strength and plays a significant role in walking and fall prevention (44, 45).

This study demonstrated that tailored exercise and nutritional interventions in senior daycare centers can significantly improve toe grip strength and hallux strength in older adults. These improvements in muscle strength could potentially lead to better health outcomes, such as enhanced balance and reduced fall risk, which are crucial for maintaining independence and overall well-being in older adults. Regular physical exercise, particularly resistance training, helps preserve and increase muscle mass and strength in older adults by promoting muscle protein synthesis and enhancing neuromuscular function. This is crucial for counteracting the effects of sarcopenia associated with aging (63). Adequate protein intake is essential for maintaining muscle mass and function, and protein supplementation ensures that older adults meet their daily protein requirements, aiding in muscle repair and growth (64). When combined with exercise, it can significantly enhance muscle strength and physical performance (65). Additionally, exercises focusing on balance and functional movements improve stability and reduce the risk of falls (66). Strengthening the muscles in the legs and feet directly enhances an older adult’s ability to perform daily activities safely and independently (67).

This study has significant strengths in evaluating methods to improve the health outcomes of older adults through tailored exercise and nutritional interventions in senior daycare centers. By presenting a program that is practically applicable, the study demonstrates that its findings can be implemented in real-world settings with tangible impacts with very old participants. This study had several limitations. First, owing to ethical considerations, control group centers run exercise programs that may have influenced the increase in toe strength. In this study, the control group engaged in exercise programs not related to our study during the intervention period. This limitation is a common feature of community intervention research. Despite this, our regression analysis showed a significant increase in hallux strength in the nutrition + exercise group and exercise group compared with the control group. Second, the retention rate was relatively low because of participants’ dropout. Many participants left the study because of health problems and cognitive decline, which is typical in a group setting such as senior daycare centers. Thus, our results must be interpreted in light of these group characteristics. In addition, as some participants had cognitive decline, we could not collect data on the nutritional index and well-being index through questionnaires. Therefore, we recommend the use of simple objective measures for studies involving older individuals aged ≥ 80 years. Future studies may benefit from larger sample sizes with diverse populations.

The findings of this study highlight the feasibility of tailored nutrition and exercise programs for older adults at daycare centers. The program demonstrated significant improvements in toe grip strength among participants. Continued implementation may enhance the balance and strength of participants, further preventing future falls. Training staff at these centers can improve the quality of care provided. Policy development should support broader access to these programs across more daycare centers. Additionally, using simple and objective measurement tools to assess the health status of older adults with cognitive decline is crucial. By adopting these approaches, senior daycare centers can significantly enhance the health, safety, and quality of life of older adults, ultimately reducing healthcare costs and improving the effectiveness of care.

## 5 Conclusion

The most significant finding of this study was the demonstration that integrating customized exercise and nutritional interventions into senior daycare centers can significantly improve toe grip strength and hallux strength in older adults. These improvements in muscle strength could potentially lead to better health outcomes, such as enhanced balance and reduced fall risk, which are crucial for maintaining independence and overall well-being in older adults. This study successfully addressed the need to develop comprehensive health promotion programs that are accessible and practical in older adults care settings. The significant improvements in toe grip strength and overall muscle health among participants demonstrate the potential of these interventions to reduce the risk of falls, improve mobility, and enhance the quality of life for older adults. Furthermore, this study highlights the necessity of developing staff training and support policies in senior daycare centers to ensure the consistent and effective application of these programs. By focusing on both physical and nutritional aspects, this study provides a comprehensive approach to older adult care. The results of this study advocate for further research into such interventions, ultimately contributing to the promotion of health among older adults.

## Data availability statement

The original contributions presented in the study are included in the article/supplementary material, further inquiries can be directed to the corresponding authors.

## Ethics statement

The studies involving humans were approved by Institutional Review Board of Hallym University. The studies were conducted in accordance with the local legislation and institutional requirements. Written informed consent for participation in this study was provided by the participants’ legal guardians/next of kin. Written informed consent was obtained from the individual(s) for the publication of any potentially identifiable images or data included in this article.

## Author contributions

JS: Data curation, Formal analysis, Investigation, Resources, Writing – original draft. JL: Conceptualization, Investigation, Methodology, Project administration, Supervision, Validation, Writing – review & editing. HL: Data curation, Investigation, Writing – review & editing. SP: Conceptualization, Data curation, Formal analysis, Funding acquisition, Methodology, Project administration, Resources, Supervision, Validation, Writing – review & editing. DS: Conceptualization, Data curation, Funding acquisition, Investigation, Methodology, Project administration, Resources, Supervision, Validation, Writing – review & editing.
